# Intranasal monoclonal antibodies to mugwort pollen reduce allergic inflammation in a mouse model of allergic rhinitis and asthma

**DOI:** 10.3389/fimmu.2025.1595659

**Published:** 2025-07-11

**Authors:** Kairat Tabynov, Igor Nedushenko, Elmira Tailakova, Akbota Sergazina, Turlan Bolatbekov, Gleb Fomin, Tair Nurpeissov, Utsav Vaghasiya, Nikolai Petrovsky, Anton Demyanov, Yuri Lebedin, Kaissar Tabynov

**Affiliations:** ^1^ International Center for Vaccinology, Kazakh National Agrarian Research University (KazNARU), Almaty, Kazakhstan; ^2^ Preclinical Research Laboratory with Vivarium, M. Aikimbayev National Research Center for Especially Dangerous Infections, Almaty, Kazakhstan; ^3^ T&TvaX LLC, Almaty, Kazakhstan; ^4^ Department of General Immunology, Asfendiyarov Kazakh National Medical University (KazNMU), Almaty, Kazakhstan; ^5^ Vaxine Pty Ltd, Adelaide, SA, Australia; ^6^ Xema Oy, Lappeenranta, Finland; ^7^ Republican Allergy Center, Research Institute of Cardiology and Internal Medicine, Almaty, Kazakhstan

**Keywords:** allergen-specific monoclonal antibody, *Artemisia vulgaris*, intranasal immunotherapy, airway inflammation, allergic rhinitis, asthma, Th2 cytokines, IgE-blocking antibody

## Abstract

**Introduction:**

Allergen-specific monoclonal antibodies (mAbs) have recently emerged as promising tools in allergy therapy, particularly for patients who do not respond adequately to allergen-specific immunotherapy (AIT). While previous studies have explored systemic delivery routes, the efficacy of local intranasal administration of allergen-specific mAb remains largely unexplored. *Artemisia vulgaris* pollen is among the top global aeroallergens, strongly associated with seasonal allergic rhinitis and asthma.

**Methods:**

Hybridoma-derived murine IgG_1_ mAb specific to *A. vulgaris* pollen extract were generated and screened *in vitro* for their ability to block both mouse and human IgE binding to crude pollen extract and its major allergen, Art v 1. A lead mAb candidate was selected for *in vivo* evaluation using a BALB/c mouse model of allergic airway inflammation. The mAb was administered intranasally one hour prior to each of three consecutive allergen challenges. Clinical symptoms, airway hyperresponsiveness (AHR), lung cytokine profiles, and histopathological changes in nasal and lung tissues were assessed.

**Results:**

Five IgG_1_ mAb recognising *A. vulgaris* extract were generated, with clone XA19 being the most potent, with high-affinity binding and IgE-blocking activity for both pollen extract (18-22% inhibition) and recombinant Art v 1 protein (52% inhibition). Intranasal pretreatment with XA19 prior to allergen challenge in pre-sensitised mice resulted in significant suppression of the ear swelling response, rhinitis symptoms, AHR, and lung and nasal turbinate inflammation. Pulmonary levels of Th2 cytokines (IL-4 and IL-5) were markedly reduced in mAb-pretreated mice, while total serum IgE levels remained largely unaffected.

**Conclusion:**

Intranasal delivery of allergen-specific mAbs represents a novel, non-invasive strategy to prevent both upper and lower airway allergic inflammation. Our findings establish proof-of-concept for this approach and warrant further development.

## Introduction

Allergen-specific immunotherapy (AIT) is an effective treatment strategy for allergic rhinitis caused by aeroallergens such as plant pollen, animal dander, or house dust. This approach is typically employed when conventional pharmacological therapies (e.g., antihistamines) are insufficient to control symptoms. AIT involves the repeated administration of gradually increasing doses of the allergen, followed by maintenance therapy over several years. The primary goal is to induce immunological tolerance and reduce hypersensitivity by modulating the immune response, which in many cases leads to long-term symptom remission (1, 2).

While the clinical benefits of AIT are well-established, the precise biological mechanisms driving its effects remain incompletely understood and subject to ongoing research. One hypothesis suggests that therapeutic effects are mediated by the suppression of effector T cell responses through the induction of allergen-specific regulatory T cells (3), T cell anergy (4, 5), and a shift from Th2 to Th1 immune polarization (6). However, attempts to replicate this mechanism using T cell epitope-based peptide vaccines, such as in cat allergy, failed during phase III clinical trials (7).

An alternative hypothesis emphasizes the role of allergen-specific IgG antibodies induced by AIT, which may compete with IgE for allergen binding, thereby preventing activation of effector cells and immediate hypersensitivity responses (8–11). Although increased allergen-specific IgG levels do not consistently translate into clinical benefit (12), a stronger association has been reported between clinical outcomes and the ability of IgG to block allergen-IgE binding (8, 13, 14). These findings highlight the critical importance of enhancing the IgG/IgE ratio to reduce allergic symptoms.

One prominent example of this approach is the successful treatment of cat allergies using monoclonal antibodies that block the interaction of the major cat allergen Fel d 1 with IgE. Studies have demonstrated that a single subcutaneous injection of these allergen-blocking antibodies significantly reduced clinical symptoms, achieving an effect comparable to that of multi-year AIT as early as day eight post-treatment (15).

Importantly, it has also been shown that clinical improvement in patients with grass pollen-induced allergic rhinitis correlates more strongly with local (nasal mucosal), rather than systemic, levels of blocking IgG antibodies induced by AIT (16). This key observation underscores the crucial role of local immune responses in desensitization and highlights the potential of intranasal delivery as a promising alternative to systemic administration of allergen-blocking antibodies.

Building on this key observation, we present the first *in vivo* evidence demonstrating the efficacy of intranasal monoclonal antibody (mAb) therapy targeting *Artemisia* pollen - a major global aeroallergen (17) - in reducing features of allergic rhinitis and asthma. IgG_1_ antibodies were selected based on their ability to block IgE binding to *A. vulgaris* extract and its major allergen, Art v 1, and were tested in a validated BALB/c mouse model of allergic airway inflammation (18, 19).

## Materials and methods

### Generation of murine mAb against *Artemisia vulgaris* pollen extract allergen

BALB/c mice (6–8 weeks old, female) were immunized intraperitoneally with 1000 PNU of *A. vulgaris* natural pollen extract (Burly, Almaty, Kazakhstan) emulsified in complete Freund’s adjuvant, followed by three booster injections at two-week intervals with the same antigen dose in incomplete Freund’s adjuvant. Three days after the final booster, spleens were harvested and splenocytes were fused with SP2/0 myeloma cells using polyethylene glycol (PEG 1500, Roche) according to standard hybridoma technology, as previously described (20). Hybridomas secreting allergen-specific IgG_1_ antibodies were screened by ELISA using plates coated with either *A. vulgaris* pollen extract or the recombinant major allergen Art v 1. Positive clones were subsequently subcloned by limiting dilution to ensure monoclonality.

### Analysis of antibody binding by direct ELISA

To assess the binding specificity of mAb, direct ELISA was performed using either recombinant Art v 1 protein or *A. vulgaris* pollen extract as the coating antigen. The recombinant Art v 1 (rArt v 1) protein was obtained as previously described (21). Antigens were diluted to a final concentration of 100 PNU/mL (*A. vulgaris* pollen extract) or 1 µg/mL (rArt v 1 protein) in carbonate buffer (pH 9.5), and 100 µL of the solution was added to each well of a high-binding polystyrene microplate (KHB, China). Plates were incubated overnight at 4 °C. Following removal of the coating solution by vacuum, wells were washed once with ELISA washing buffer (0.1% Tween-20, Serva, Germany, in 0.9% NaCl, Merck, Germany) and subsequently blocked with blocking buffer (0.1 M phosphate buffer containing 0.9% NaCl and 0.5% hydrolyzed casein) for 2 h at room temperature (RT). After blocking, plates were dried at RT for 48 h and stored until use. Hybridoma culture supernatants or purified mAb were serially diluted in ELISA dilution buffer (0.1 M phosphate buffer containing 0.9% NaCl and 0.1% hydrolyzed casein). Aliquots of 100 µL were added to each well and incubated for 30 min at 37 °C. Wells were then washed three times with washing buffer, followed by incubation with horseradish peroxidase (HRP)-conjugated sheep anti-mouse IgG antibodies (Cat# AS302-HRP, Xema, Finland) for 30 min at 37 °C. After five washes, TMB substrate solution (Cat# R055, Xema) was added to each well and incubated for 15 min at RT. The reaction was terminated by the addition of 5% sulfuric acid, and absorbance was measured at 450 nm using a HiPo microplate reader (Biosan, Latvia). Only clones demonstrating binding to both the rArt v 1 protein and natural pollen extract were selected for further characterization.

### Competition of mAb with human IgE by reverse binding ELISA

To assess the ability of mAb to inhibit the interaction between human IgE and *A. vulgaris* allergens, a reverse binding ELISA was performed using ready-to-use components from a specific IgE detection kit (#K200S, Xema). The assay was conducted following a modified protocol. At the first step, 100 µL of serum containing high levels (Class III) of mugwort-specific IgE from a single allergic patient (nArt v 1-specific IgE [w231] level: 5.21 kU/L, determined by immunofluorescence on a Phadia 250 analyzer, Sweden) was added to microplate wells pre-coated with anti-human IgE monoclonal antibodies. The plate was incubated for 60 min at 25 °C with continuous shaking on a platform shaker (ELMI, Latvia). After three washes with ELISA washing buffer (0.1% Tween-20 in 0.9% NaCl), 50 µL of biotinylated natural mugwort allergen was added to each well along with 50 µL of one of the following: ELISA buffer (negative control) or purified anti-mugwort pollen mAb. The plate was incubated for another 60 min at 25 °C under the same shaking conditions, followed by three additional washes. In the third step, 100 µL of streptavidin–polymerized HRP conjugate (working dilution) was added to each well and incubated for 30 min at 25 °C without shaking. After five final washes, 100 µL of TMB substrate (Cat# R055, Xema) was added and incubated for 15 min at RT. The enzymatic reaction was stopped by adding 5% sulfuric acid, and optical density (OD) was measured at 450 nm using a HiPo microplate reader (Biosan, Latvia). All samples were assayed in triplicate. The percentage of inhibition of IgE-allergen binding was calculated using the following formula:


$$\text{Inhibition}\left(\%\right)=\frac{\text{ODsample​}-\text{ODcontrol}}{\text{ODcontrol​ }}\times 100$$

### Competition of mAb with mouse IgE by reverse binding ELISA

Ninety-six-well microplates (Thermo Fisher Scientific, Waltham, MA, USA) were coated with 100 µL per well of a capture antibody specific for mouse IgE (ELISA MAX™ Standard Set Mouse IgE, BioLegend) and incubated overnight at 2–8 °C. The next day, the plates were blocked with 200 µL of blocking buffer per well and incubated at RT for 1 h on a PST-60HL thermal shaker (BIOSAN, Latvia). After blocking, wells were washed four times with washing buffer. Mouse serum samples - obtained from either A. *vulgaris*-sensitized mice (as previously described (18, 19)) - were diluted 1:5 in ELISA Assay Diluent. Subsequently, 100 µL of each diluted serum sample was added to the wells and incubated for 1.5–2 h at RT with shaking. After four additional washes, 50 µL of biotinylated natural mugwort allergen or rArt v 1 protein was added to each well, along with 50 µL of either ELISA buffer (negative control) or purified anti-mugwort pollen mAb. The remaining steps, including the detection procedure and calculation of the percentage inhibition of IgE-allergen binding, were performed as described previously for the human IgE assay. For subsequent *in vivo* studies, only those mAb demonstrating the strongest and most consistent inhibition of mouse IgE binding to both *A. vulgaris* extract and rArt v 1 were selected. Among these, the clone XA19 showed the most potent allergen-blocking activity and was subsequently sequenced (GenBank accession numbers BankIt2947155 Seq1 PV483362 and BankIt2947155 Seq2 PV483363).

### Modelling of XA19 mAb interaction with Art v 1

The gene sequence for the synthetic construct Art v 1 major *Artemisia* pollen allergen was retrieved from the NCBI GenBank database (accession number: PQ223694.1). The Open Reading Frame (ORF) was identified using the NCBI ORF Finder tool, yielding the amino acid sequence:

MGSSHHHHHHSSGLVPRGSHMAGSKLCEKTSKTYSGKCDNKKCDKKCIEWEKAQHGACHKREAGKESCFCYFDCSKSPPGATPAPPGAAPPPAAGGSPSPPADGGSPPPPADGGSPPVDGGSPPPPSTH. To obtain the mature protein sequence for structural modelling of Art v 1, the N-terminal His-tag & linker sequence (MGSSHHHHHHSSGLVPRGSH) were removed. The resulting sequence was used for subsequent protein modelling analyses. To determine the molecular interactions driving the binding of XA19 to Art v 1, structural models of XA19 fab region and of Art v 1 were generated using AlphaFold3 and docking of the structural models was performed using the AlphaFold webserver. An all-atom molecular dynamic simulation of the XA19– Art v 1 complex was conducted using GROMACS [v2022.6 GPU], with the production run extending over 100 ns. The system was propagated using the leap-frog integrator with a 2 fs timestep, totalling 50 million steps. Coordinate and velocity outputs to the.trr file were suppressed to reduce file size, while compressed coordinates were saved every 10 ps. Energies and log files were also recorded at 10 ps intervals. All bonds involving hydrogen atoms were constrained using the LINCS algorithm to enable the use of a 2 fs timestep. Electrostatic interactions were treated using the Particle Mesh Ewald (PME) method with a 1.2 nm cutoff and van der Waals interactions employed a force-switch scheme between 1.0 and 1.2 nm. Temperature was maintained at 298 K using a velocity-rescaling thermostat, and isotropic pressure coupling at 1.0 bar was applied using the C-rescale barostat with a compressibility of 4.5 x 10–^5^ bar^-1^. Periodic boundary conditions were applied in all three spatial dimensions, and the simulation continued from the equilibrated NPT ensemble without reinitialization of velocities. Detailed residue-residue interaction analysis was carried out for both the variable light (VL) and variable heavy (VH) chains of the Fab in complex with Art v 1.

### Purification of mAbs by protein G affinity chromatography

mAbs were purified from hybridoma culture supernatants by affinity chromatography using Protein G-agarose Resin 4 (Agarose Bead Technology, Spain), following the manufacturer’s protocol. Briefly, clarified supernatants were filtered through a 0.22 µm membrane and loaded onto a pre-equilibrated Protein G column. After binding, the column was washed extensively with phosphate-buffered saline (PBS, pH 7.4) to remove unbound proteins. Bound antibodies were eluted using 0.1 M glycine-HCl buffer (pH 2.7) and immediately neutralized with 1 M Tris-HCl (pH 9.0). Eluted fractions were analyzed by absorbance at 280 nm, and those containing IgG were pooled, dialyzed against PBS, and stored at –20 °C until use.

### Sensitization and allergen challenge in mice with pretreatment using mAb

**Figure 1:**
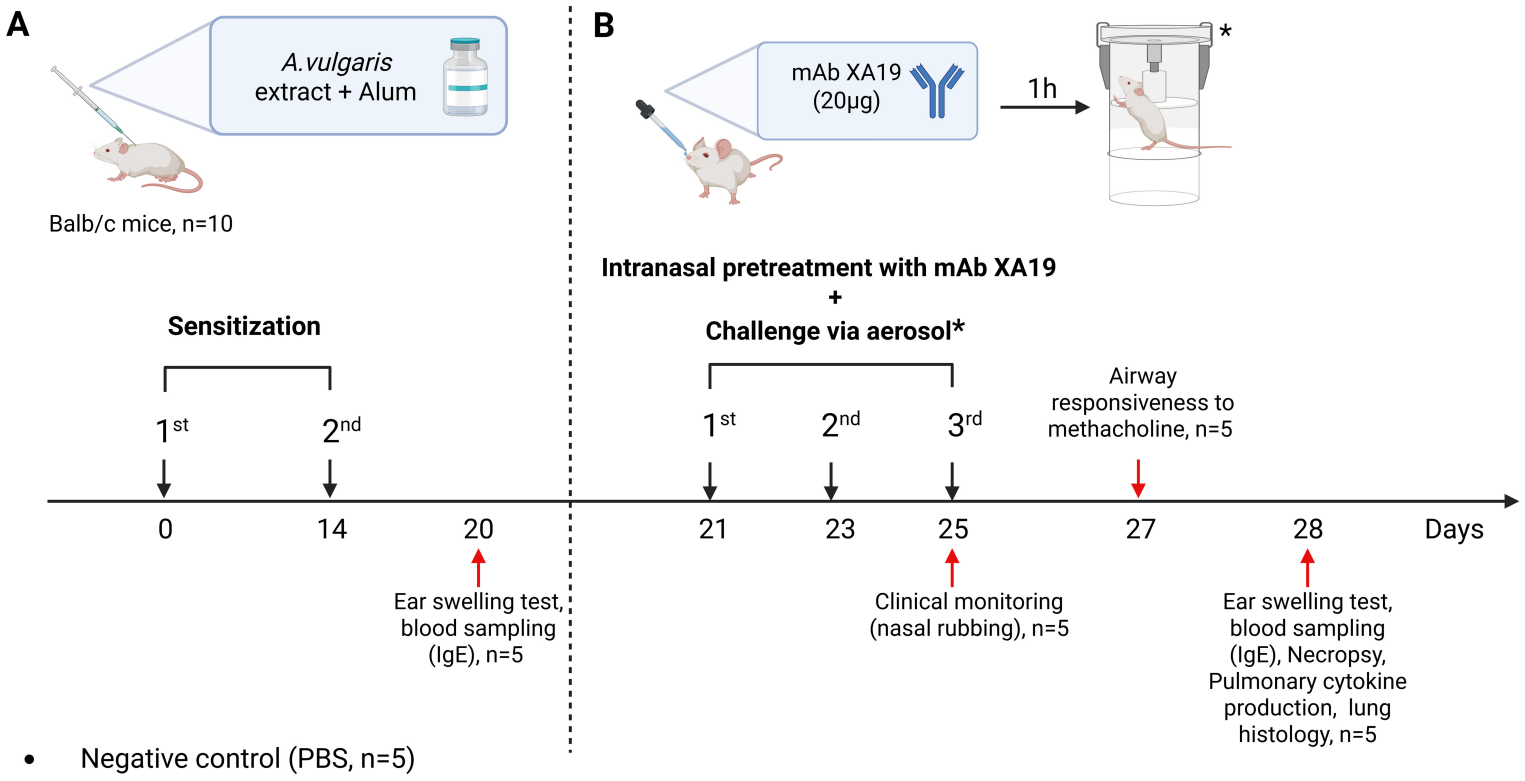
Study design. The schematic illustration shows the sensitization of mice with *Artemisia vulgaris* pollen extract **(A)**, intranasal pretreatment with the monoclonal antibody XA19 and following subsequent allergen challenge **(B)**.

A well-established murine model was used to reproduce allergic airway inflammation through intraperitoneal (i.p.) sensitization followed by aerosol and intranasal allergen challenge, as previously described (18, 19). Briefly, specific pathogen-free (SPF) male BALB/c mice aged 8–12 weeks (n = 5 per group; 15 mice in total) were randomly divided into three groups (**Figure 1**). Mice in the sensitized groups (mAb treatment and positive control) received two i.p. injections of *A. vulgaris* pollen extract (Burly, Kazakhstan) at 14-day intervals (days 0 and 14). Each injection contained 1000 PNU in 200 µL PBS, adsorbed onto aluminum hydroxide (1 mg Al³^+^ per mouse). Negative control mice (n = 5) received an equal volume (200 µL) of PBS with alum. On day 21, all mice underwent allergen challenge performed three times at 48-h intervals (on days 21, 23, and 25). Each challenge included: inhalation exposure to *A. vulgaris* pollen extract aerosol (1000 PNU per group), delivered using a previously described whole-body exposure method (19), and intranasal administration of *A. vulgaris* extract (200 PNU in 20 µL PBS) or PBS (negative control) under light ketamine-xylazine anesthesia. Anesthesia was induced via i.p. injection of ketamine (50 mg/kg) and xylazine (10 mg/kg) in sterile phosphate-buffered saline. To assess the therapeutic potential of mAbs, mice in the pretreatment group received 20 µg of the XA19 mAb (in 20 µL PBS; approximately 1 mg/kg per dose) via intranasal administration under anesthesia, one h prior to each allergen challenge. Clinical monitoring was performed during the third nebulization challenge to assess the severity of allergic rhinitis, with a focus on nasal rubbing. On day 27, airway responsiveness to methacholine or PBS was assessed. On the final day (day 28), mice underwent: an ear swelling test, blood sampling for measurement of total and allergen-specific IgE levels, necropsy for lung tissue collection, followed by cytokine quantification and histopathological analysis to assess allergic inflammation.

### Quantification of total and allergen-specific IgE by ELISA

Total and allergen-specific IgE levels were measured using enzyme-linked immunosorbent assay (ELISA). Total IgE was quantified using the ELISA MAX™ Standard Set Mouse IgE kit (BioLegend) according to the manufacturer’s instructions, with results expressed in µg/mL.

To quantify allergen-specific IgE, 96-well microplates (Thermo Fisher Scientific, Waltham, MA, USA) were coated with 100 µL per well of anti-mouse IgE capture antibody (from the same ELISA kit) and incubated overnight at 2–8 °C. On the following day, plates were blocked with 200 µL/well of blocking buffer and incubated for 1 h at RT on a thermal shaker (PST-60HL, BIOSAN), followed by four washes with wash buffer. Mouse serum samples were diluted 1:10 in assay diluent and added to the wells (100 µL/well), followed by incubation for 2 h at RT with shaking. After four washes, 100 µL of biotinylated *A. vulgaris* allergen extract were added to the respective wells and incubated for 1 h at RT with shaking. Biotinylation of allergen extracts was performed using the EZ-Link™ NHS-Biotin kit (Thermo Fisher Scientific, Rockford, IL, USA), which labels primary amines while preserving allergenic epitopes. Plates were washed five times before the addition of 100 µL/well of TMB substrate solution (BioLegend, USA). After a 15 min incubation at RT, the enzymatic reaction was stopped by adding 100 µL of 2.5 M H_2_SO_4_. OD was measured at 450 nm using a Chromate 4300 microplate reader (Awareness Technologies, USA).

### Ear swelling test

To assess local allergic hypersensitivity, mice were intradermally injected into the right auricle with 10 µL of the *A. vulgaris* pollen extract (100 PNU per mouse). Mice in the negative control group received 10 µL of PBS instead. After 1.5–2 h, auricular thickness was measured using an electronic digital micrometer (MCC-25 DSWQ0-100II, China). The degree of ear swelling was calculated as the difference in thickness (in mm) between the allergen-injected right ear and the PBS-injected left ear.

### Assessment of nasal rubbing in mice

Following the third nebulization challenge with either allergen or PBS (n = 5 per group), mice were clinically observed for 5 min to assess nasal rubbing frequency, as previously described (22). Observations began with the negative control group (PBS exposure). After each allergen administration, the chamber was thoroughly washed and dried before proceeding with the next group to prevent cross-contamination. Clinical assessments were performed by staff members blinded to the group assignments and study protocol.

### Assessment of airway hyperresponsiveness

Airway hyperresponsiveness (AHR) was evaluated using a whole-body plethysmography (WBP-M) system (Shanghai TOW Intelligent Technology Co. Ltd., Shanghai, China), following the protocol described by Hamelmann et al. (23). Mice were individually placed into the plethysmography chambers and exposed to aerosolized methacholine (25 mg/mL) or phosphate-buffered saline (PBS, negative control) for 5 min. Airway resistance was assessed by measuring the enhanced pause (Penh), a dimensionless parameter indicative of bronchoconstriction. Data acquisition and Penh calculation were performed using ResMass software version 1.4.2 (TOW, China).

### Lung tissue cytokine quantification by ELISA

Following necropsy, the left lung of each mouse was harvested and processed for cytokine analysis. Tissue samples were placed in 1 mL of Dulbecco’s Modified Eagle Medium (DMEM) and homogenized using a TissueLyser II instrument (QIAGEN, Germany) at 300 oscillations per min for 60 s. The resulting homogenates were centrifuged at 5000 × g for 15 min at 4 °C, and the supernatants were collected and stored at −70 °C until analysis. Proinflammatory cytokine levels, including IL-4, IL-5, IL-13, IL-17, TNF-α, and IFN-γ, were measured in the lung homogenate supernatants using ELISA MAX™ Deluxe Set Mouse kits (BioLegend, San Diego, CA, USA), following the manufacturer’s instructions. Cytokine concentrations were calculated based on standard curves and expressed in pg/mL.

### Histological analysis of mouse nasal turbinates and lung tissue

Following euthanasia, mouse nasal turbinates and lungs were excised, rinsed in distilled water, and fixed in 10% neutral-buffered formalin for 7–10 days. After fixation, tissues underwent a standard dehydration protocol consisting of sequential immersion in four changes of 100% isopropyl alcohol, followed by two changes of xylene. Nasal turbinates were decalcified in 20% EDTA-Na solution for 3 days prior to paraffin embedding. Tissues were then infiltrated with four changes of paraffin and embedded into histological blocks. Serial sections of 5 µm thickness were cut using a microprocessor-controlled rotary microtome (MZP-01, KB Tekhnom, Russia). The sections were deparaffinized in two changes of xylene and rehydrated through a graded ethanol series (96%, 80%, and 70%). Staining was performed with hematoxylin (#05-002, BioVitrum, Russia) and eosin (#C0362, DiaPath, Italy), followed by dehydration through ascending concentrations of ethanol (70%, 80%, and 96%) and two final changes of xylene. Coverslips were mounted using Bio Mount synthetic mounting medium (#2813, Bio Optica, Italy). Histological slides were examined using an Mshot MF52-N microscope (China) equipped with an MShot MS23 digital camera. Images were acquired at 100× magnification using the MShot Image Analysis System (China), and selected structures were also examined under oil immersion at 1000× magnification. Calibration was performed using a standardized micrometric scale, and all morphometric measurements were reported in µm. Histopathological changes in the lungs nasal tubinates were assessed using a semiquantitative scoring system described previously (19). The criteria used for scoring are summarized in **Table 1**. Histological assessments were performed blinded to treatment groups.

**Table 1:** Histopathological scoring system for mouse lung and nasal turbinate tissues.

Evaluated trait	Points for the evaluated trait
Lung	
Perivascular/peribronchial inflammation	0 – no changes; 1 – moderate inflammation; 2 – pronounced inflammation; 3 – severe inflammation
Presence of eosinophils in foci of perivascular/peribronchial inflammation	0 – absent; 1 – single eosinophils in the field with magnification (x1000); 2 – multiple eosinophils in the field with magnification (x1000)
Metaplasia of the Goblet cells in the bronchi	0 – absent; 1 – several Goblet cells are present in one or two bronchiolar profiles; 2 – numerous Goblet cells are present in bronchioles
Maximum score	7
Nasal Turbinate	
Epithelial necrosis	0 – no changes; 1 – moderate inflammation; 2 – pronounced inflammation; 3 – severe inflammation
Goblet cell hyperplasia	0 – no changes; 1 – moderate inflammation; 2 – pronounced inflammation; 3 – severe inflammation
Lymphocytic infiltration	0 – no changes; 1 – moderate inflammation; 2 – pronounced inflammation; 3 – severe inflammation
Maximum score	9

### Animal housing and ethical compliance

All animal studies were carried out at the certified vivarium facility of the M. Aikimbayev National Scientific Center for Especially Dangerous Infections (NSCEDI), under the Ministry of Health of the Republic of Kazakhstan. SPF BALB/c mice were housed, maintained, and fed in accordance with previously validated procedures (18, 19). Experimental protocols were reviewed and approved by the Institutional Animal Care and Use Committee (IACUC) at NSCEDI (Approval No. 16, dated October 31, 2022), and all procedures complied with national and international standards for the ethical use of animals in research. Humane endpoints were implemented based on IACUC-approved welfare scoring systems to guide timely euthanasia. Prior to terminal sample collection, mice were humanely euthanized under deep anesthesia induced by intraperitoneal administration of ketamine (100 mg/kg) and xylazine (40 mg/kg), followed by cervical dislocation to ensure death.

The use of human serum samples in this study was approved by the Local Ethics Committee of S.D. Asfendiyarov Kazakh National Medical University (Approval No. 14 (150), dated April 26, 2024). Written informed consent was obtained from the donor prior to sample collection.

### Statistical analysis

All statistical analyses and data visualizations were performed using GraphPad Prism version 9.0 (GraphPad Software, San Diego, CA, USA). Comparisons of antibody levels, ear swelling measurements, airway responsiveness, lung cytokine profiles, and histopathological scores among experimental groups were conducted using one-way ANOVA followed by Tukey’s multiple comparisons test. A p-value of < 0.05 was considered statistically significant. All graphical data are presented as mean ± standard error of the mean (SEM).

## Results

### High-Affinity mAb Effectively Inhibits Human and Murine IgE Binding to *A vulgaris* Extract and Its Major Allergen Art v 1

A total of five hybridoma clones (XA15, XA19, XA36, XA37, and XA40) producing IgG_1_ antibodies specific to *A. vulgaris* pollen extract were successfully generated and purified, with final antibody concentrations ranging from 1.0 to 2.5 mg/mL. Of these, only two clones - XA19 and XA15 -recognized both the natural pollen extract and the recombinant major allergen Art v 1 in direct binding ELISA. However, only the culture supernatant of clone XA19 demonstrated IgE-blocking activity in the reverse ELISA, achieving 18% inhibition of human IgE binding to *A. vulgaris* extract. In murine IgE-blocking ELISA, purified XA19 mAb reduced IgE binding to the natural pollen extract and Art v 1 by 22% and 52%, respectively. Based on its dual specificity and superior IgE-blocking capacity across both human and murine models, clone XA19 was selected for subsequent *in vivo* studies.

### Intranasal Pretreatment with Allergen-Specific mAb Attenuates Local Allergic Response with Minor Impact on IgE Levels

**Figure 2:**
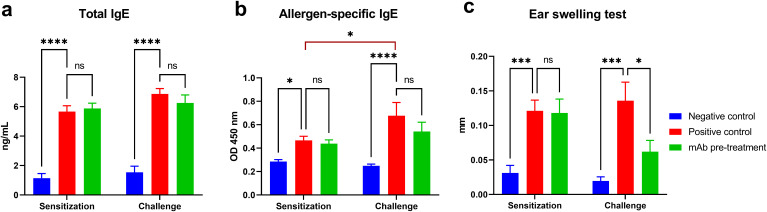
Evaluation of humoral and local allergic responses in sensitized mice following allergen challenge with or without mAb intranasal pretreatment. The figure shows the levels of total IgE **(a)**, allergen-specific IgE **(b)**, and results of the ear swelling test **(c)** in mice after sensitization and allergen challenge. Mice in the mAb pretreatment group received intranasal administration of anti-*Artemisia vulgaris* pollen extract monoclonal antibodies (mAb) under light ketamine-xylazine anesthesia, 1 h prior to each allergen challenge. The challenge was performed via intranasal instillation and aerosolized nebulization of *A*. *vulgaris* pollen extract. Total IgE concentrations are presented in ng/mL, while allergen-specific IgE levels are expressed as optical density values measured at 450 nm. Statistical analysis of IgE levels between groups was conducted using Tukey’s multiple comparisons test. Differences were considered statistically significant at *P*<0.05. *P < 0.05, ***P < 0.001, and ****P < 0.0001.

Successful sensitization of mice to *A. vulgaris* pollen was achieved in both experimental groups (positive control and mAb pretreatment), as evidenced by a significant accumulation of total and allergen-specific IgE, as well as pronounced sensitization according to the ear swelling test compared to the negative control group (**Figure 2**). Following triple allergen challenge, mice in the positive control group exhibited a marked increase in allergen-specific IgE levels (vs. sensitization), and other laboratory parameters also showed a trend toward elevation. In contrast, in the mAb pretreatment group, although a moderate increase in total and allergen-specific IgE was observed compared to post-sensitization levels, these values remained slightly lower than in the positive control group. Notably, the ear swelling test in the mAb pretreatment group did not follow the same upward trend. Instead, a decrease in this parameter was recorded compared to the sensitization phase and, more significantly, a substantial reduction relative to the positive control group.

### Allergen-specific mAb intranasal pretreatment significantly alleviates *A vulgaris*-induced allergic airway inflammation

**Figure 3:**
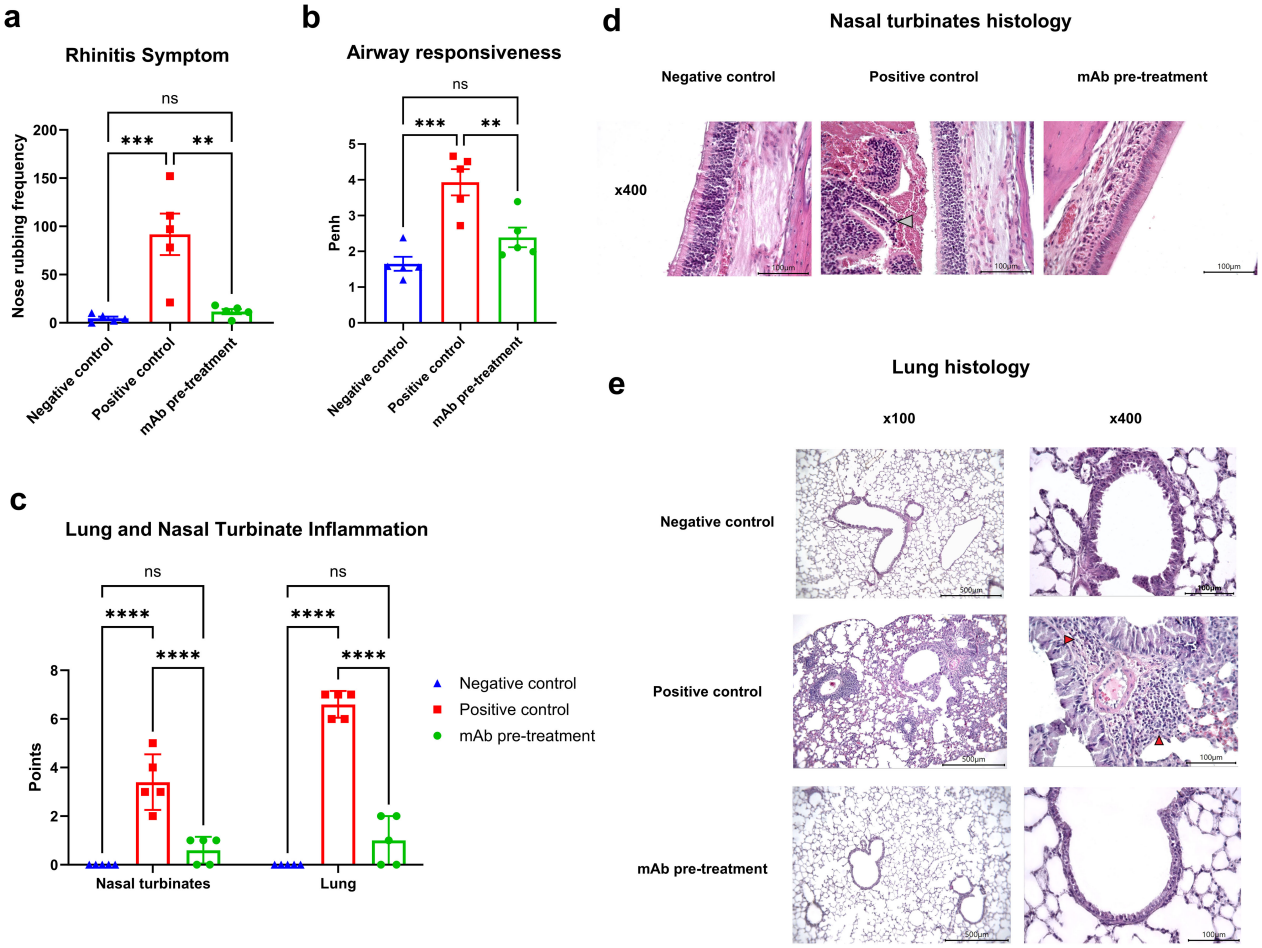
Efficacy of intranasal pretreatment with anti-*A. vulgaris* pollen mAb in reducing clinical and pathological respiratory signs in sensitized mice after allergen challenge. Clinical signs of allergic rhinitis were assessed by quantifying the number of nasal rubbing events after the third allergen challenge **(a)**. Bronchial asthma severity was evaluated based on airway hyperresponsiveness using whole-body plethysmography following methacholine exposure, presented as Penh values **(b)**. Allergen-induced inflammation of the nasal turbinates and lungs was scored using a semiquantitative histological grading system **(c)**. Representative histological images of nasal turbinates from each experimental group are shown at 400× magnification **(d)**. Notably, nasal cavity debris in the positive control group is indicated by a grey arrow. Panel **(e)** displays representative lung histology images at both 100× and 400× magnification for each group. Statistical differences between groups were analyzed using Tukey’s multiple comparisons test. NS = not significant. P < 0.05 was considered statistically significant. **P<0.01, ***P<0.001, ****P<0.0001.

At this stage, we evaluated clinical manifestations of rhinitis and bronchial asthma, as well as histopathological alterations in the nasal turbinates and lung tissue, in *A. vulgaris*-sensitized mice with or without mAb intranasal pretreatment prior to three consecutive allergen challenges. mAb pretreatment markedly reduced both clinical symptoms - reflected by a significant decrease in nasal rubbing episodes - and histopathological signs of rhinitis when compared to the positive control group (**Figures 3a, c**). Histological analysis of nasal turbinates in the mAb pretreatment group revealed well-preserved respiratory epithelium, consisting predominantly of stratified, ciliated columnar epithelial cells (**Figure 3d**). A small number of goblet cells were detected. The olfactory epithelium appeared as pseudostratified columnar epithelium with only minor focal epithelial necrosis. The lamina propria was intact, without signs of inflammatory cell infiltration or thickening. The submucosal glands displayed normal morphology. The mean histopathological score for nasal turbinate inflammation in this group was 0.6 out of 9, with pathological changes detected in only 3 out of 5 mice. Based on the combined clinical signs and histopathological changes in the nasal turbinates, mice in the mAb pretreatment group showed no significant differences compared to the negative control group.

In contrast, all mice (5/5) in the positive control group exhibited pronounced histopathological changes. Goblet cell hyperplasia was observed in focal regions, and the lamina propria displayed structural disruption and signs of inflammatory remodeling (**Figure 3d**). Condensed, pyknotic nuclei characteristic of karyorrhexis were present in both respiratory and olfactory epithelial cells. Large areas of epithelial desquamation were observed, along with individual leukocytes and abundant erythrocytes within the nasal cavity lumen, forming dense cellular debris masses. The mean inflammation score in this group was 3.4 out of 9.

No clinical or histopathological signs of rhinitis were observed in mice from the negative control group.

AHR, a defining feature of clinical asthma, was assessed via whole-body plethysmography in response to methacholine challenge (**Figure 3b**). Sensitized mice (positive control group) exposed to allergen challenges with *A. vulgaris* pollen exhibited pronounced AHR in response to methacholine, compared to the negative control group. In contrast, sensitized mice that received intranasal administration of anti-*A. vulgaris* mAb one hour prior to each allergen challenge showed significantly reduced AHR. The AHR levels in this group were comparable to those observed in the negative control group.

These functional outcomes were further supported by histopathological analysis of lung tissue (**Figure 3c**). Mice that received mAb intranasal pretreatment displayed significantly reduced pulmonary inflammation compared to the positive control group, with inflammatory levels comparable to those observed in the negative control group.

Histological examination of lung sections from the mAb pretreatment group revealed preserved pleural integrity without thickening or visible signs of inflammation (**Figure 3e**). Bronchial architecture remained intact, although mild goblet cell metaplasia was observed in a minority of bronchi. Low-grade, focal peribronchial lymphocytic infiltrates were detected in 3 out of 5 animals. Alveolar structures were preserved, with normal interalveolar septa, lacking any signs of thickening, inflammation, or hemorrhage. In the positive control group, the pleura also appeared preserved, without thickening or overt inflammation. Bronchial structures remained intact; however, multifocal, marked peribronchial inflammation dominated by macrophages and monocytes was noted in 4 out of 5 mice, along with moderate peribronchial infiltrates. Eosinophils were abundantly present within the inflammatory foci. Goblet cell hyperplasia was prominent in the bronchi of all animals. The alveolar architecture remained preserved, and interalveolar septa showed no signs of thickening, inflammation, or hemorrhage.

No pathological changes were detected in the lung tissues of the negative control group.

### Intranasal pretreatment with anti-*A. vulgaris* mAb reduces lung Th2-associated IL-4 and IL-5 responses following allergen challenge

**Figure 4:**
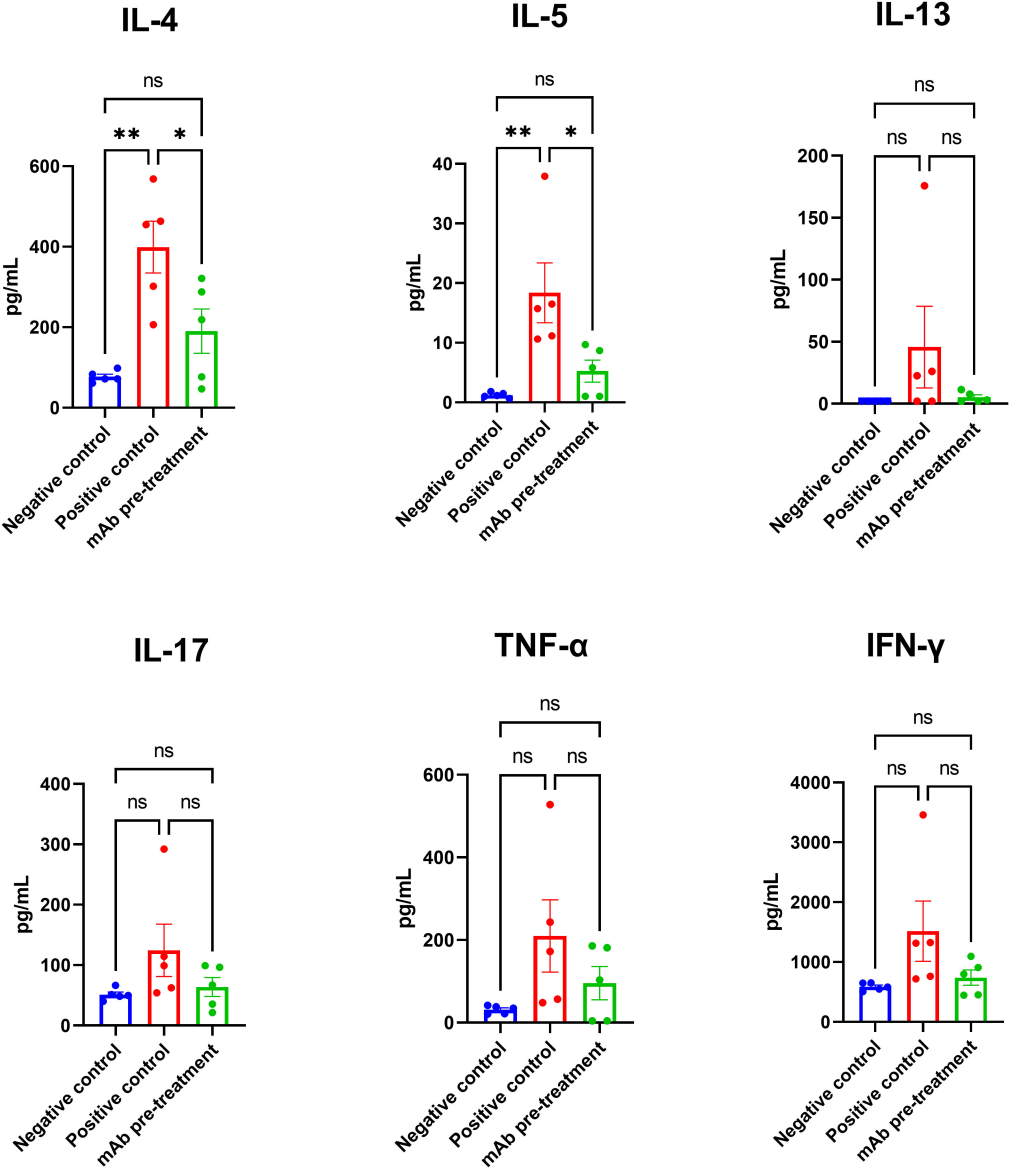
Comparison of lung cytokine profiles in sensitized mice with or without intranasal anti-*A. vulgaris* pollen mAb pretreatment following allergen challenge. Proinflammatory cytokine concentrations in lung homogenates are expressed in pg/mL. Data are presented as mean ± SEM. Statistical differences between groups were evaluated using Tukey’s multiple comparisons test. *NS* = not significant. A *P*-value < 0.05 was considered statistically significant. *P<0.05, **P<0.01.

Following allergen challenge, mice in the positive control group exhibited a marked increase in the production of key Th2-associated cytokines IL-4 and IL-5 in lung tissue compared to the negative control group (**Figure 4**). Intranasal pretreatment with anti-*A. vulgaris* mAb significantly suppressed the expression of IL-4 and IL-5 in sensitized mice, relative to the untreated positive controls. For the remaining cytokines analyzed (IL-13, IL-17, TNF-α, and IFN-γ), a trend toward elevated levels was observed in the positive control group; however, these differences did not reach statistical significance when compared with the negative control group.

### Modelling of XA19 interaction with Art v 1

**Figure 5:**
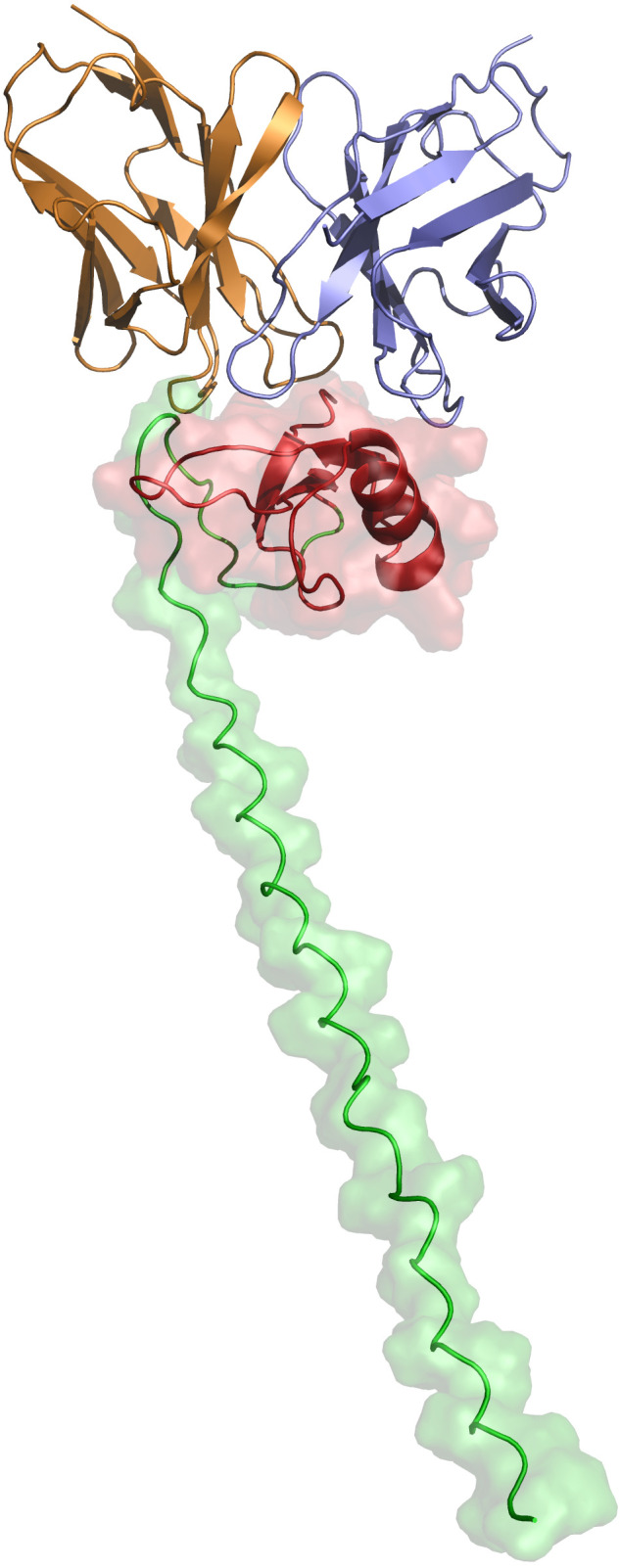
Structural representation of the predicted XA19–Art v 1 docked complex after MDS optimization. The defensin-like head region of the MPV Art v 1 antigen is depicted in red, while the tail region is shown in green. The variable heavy (VH) chain of the XA19 antibody is colored purple, and the variable light (VL) chain is represented in orange.

Art v 1, the major allergen of *A. vulgaris* pollen, has a cysteine-rich N-terminal domain that has homology to plant defensins which are small, basic peptides with a cysteine-stabilized alpha-beta (CSαβ) fold that defend plants, animals and insects against pathogens and parasites, through various mechanisms including membrane permeabilization, proteinase and amylase inhibitory activity and inhibition of protein translation (24). Elucidating the structure and mode of binding of XA19 to Art v 1 might enable better understanding of how XA19 mAb is able to inhibit Art v 1 allergenicity. As no high-resolution crystal structure of Art v 1 was available, we used AlphaFold3 to perform a structural prediction of Art v 1. This revealed Art v 1 to have a classic “head and tail” structure (**Figure 5**), as previously suggested by another modelling study (25). We next used AlphaFold to predict the XA19 Fab antigen binding domain structure and to perform blind docking of it to the predicted Art v 1 structure. GROMACS was then used to perform a molecular dynamic simulation to optimize the docked complex. The modelled complex shows both the heavy and light chains of XA19 bound the head ‘defensin-like’ domain of Art v 1 with binding of XA19 stabilising the Art v 1 structure (**Figure 5**). Interestingly, the defensin-fold of Art v 1 has been shown to form epitopes recognized by IgE antibodies from allergic patients (26). Hence XA19 may sterically hinder binding of IgE to these same defensin-domain epitopes, as suggested by our competition ELISA data. Further or in the alternative, the binding of XA19 to Art v 1 may induce a conformational change in Art v 1 that in turn induces blocking or masking of key IgE epitopes.

## Discussion

mAbs have emerged as an effective and promising tool in targeted therapy for various forms of allergy, especially in cases where traditional AIT proves insufficient. The most widely used mAb is omalizumab, a humanized IgG_1_ antibody that binds to free IgE, thereby preventing its interaction with high-affinity FcϵRI receptors on mast cells and basophils. Omalizumab has been approved for the treatment of persistent allergic asthma, chronic spontaneous urticaria, and allergic rhinitis (27, 28). Additionally, dupilumab, an anti–IL-4 receptor alpha (IL-4Rα) antibody, has demonstrated clinical efficacy in treating atopic dermatitis, eosinophilic asthma, and polysensitized allergic conditions (29), and recent clinical trials involving localized delivery of similar cytokine-targeting biologics - such as Stapokibart (anti–IL-4Rα) administered intranasally - further support the feasibility of local biologic therapy for allergic airway diseases (30). However, these mAb do not fall within the narrow definition of passive allergen immunotherapy, not being allergen-specific.

In contrast, allergen-specific mAbs have gained increasing attention as precise tools for passive AIT, aimed at neutralizing defined allergenic proteins. Notable examples include antibodies targeting Fel d 1, the major cat allergen (15), or Ara h 1 and Ara h 3, key peanut allergens (31). These mAb candidates when administered via parenteral injection have demonstrated promising efficacy in preclinical models, providing a strong foundation for advancement into clinical trials (32).

Our findings reveal, for the first time, that intranasal pretreatment with allergen-specific mAbs offers protective effects against *Artemisia* pollen, one of the most impactful airborne allergens worldwide. In particular, our mAb (clone XA19), inhibited human and murine IgE binding *in vitro* to *A. vulgaris* extract and its major allergen Art v 1. Notably, the IgE-blocking efficiency was an order of magnitude higher for Art v 1 than for the pollen extract. This is attributed to the fact that although Art v 1 is the most clinically significant allergenic component in mugwort pollen (33), it is just one of the ten known *Artemisia* pollen allergens (34). The native Art v 1 protein in natural pollen extracts reacts with IgE in sera of over 95% of patients with *Artemisia* allergy (26). In the present study, a recombinant full-length Art v 1 protein was used for mAb clone screening. Although reports suggest that the recombinant Art v 1 protein may have reduced allergenicity in human provocation tests compared to native forms (35), our previous findings strongly support the functional relevance of recombinant Art v 1 given its success as both a therapeutic antigen in murine AIT models and as a diagnostic tool for IgE quantification in ELISA (18, 19, 21).

To assess the *in vivo* efficacy of XA19 mAb, we used a validated mouse model of *Artemisia* pollen-induced allergic airway inflammation (19). Intranasal mAb pretreatment was administered one hour prior to each of three allergen exposures, delivered through both nasal instillation and inhalation. Given the exploratory nature of this study, only one dose and timing regimen were evaluated. This design was inspired by our previous study on XR10v48, an anti-SARS-CoV-2 mAb which showed potent protective activity when given as an intranasal pretreatment in a hamster COVID-19 model (36). XA19 pretreatment was protective in *A. vulgaris*-sensitized mice as confirmed by reduction in laboratory, clinical, and pathological indicators of allergic rhinitis and bronchial asthma. Although systemic total and allergen-specific IgE levels were not affected by mAb pretreatment, local allergic inflammation was markedly attenuated. This is consistent with allergen-specific IgG mAbs primarily functioning through allergen neutralization at the mucosal surface rather than through suppression of IgE production. The absence of a significant decrease in IgE levels despite strong anti-inflammatory effects suggests alternative mechanisms such as competition with IgE for allergen binding, interference with allergen uptake by mucosal dendritic cells, promotion of regulatory T cells (Tregs), or FcγRIIb-mediated inhibitory signaling via IgG-allergen immune complexes. These pathways have been described for other allergen-specific mAbs and represent important areas for future investigation. Similar protective effects have been reported in studies of Fel d 1-specific IgG antibodies, which reduced airway inflammation and basophil activation without altering IgE titers in cat-allergic individuals (37). Furthermore, in a phase 1 study of peanut allergy, anti–Ara h 2 mAbs, despite not significantly reducing IgE levels, inhibited mast cell activation and increased the allergic threshold upon challenge (38). Taken together, our current results and previous findings support the concept that allergen-specific mAbs can confer clinical benefit regardless of IgE levels, through blocking allergen-IgE interactions at the effector cell level. Intranasal mAb pretreatment led to a marked reduction in clinical symptoms of rhinitis, including a significant decrease in nasal rubbing episodes, and attenuated histopathological signs of inflammation in the nasal turbinates. These changes included preserved epithelial integrity and the absence of inflammatory cell infiltration. This indicates that the mAbs efficiently block allergen interaction with immune effector cells at the nasal mucosa, thereby preventing the initiation of local inflammatory responses. Moreover, mAb administration significantly reduced both AHR and lung inflammation, supporting a broader protective effect extending beyond the upper airway. Hence intranasal administration of *A. vulgaris-* specific mAbs effectively prevented the development of both allergic rhinitis and bronchial asthma in sensitized mice. Histological examination of the lungs revealed a decrease in peribronchial inflammation, eosinophilic infiltration, and goblet cell hyperplasia in the mAb-treated group. These improvements were paralleled by a significant reduction in the levels of the Th2-associated cytokines IL-4 and IL-5 in lung tissue, indicating suppression of Th2-mediated airway inflammation. This aligns with previous evidence highlighting the central role of IL-4 (36, 39) and IL-5 (40) in driving allergic airway inflammation and eosinophil recruitment. This is particularly significant, as intranasal mAb delivery not only suppressed local nasal allergic responses but also prevented systemic respiratory manifestations, such as AHR and pulmonary inflammation.

Despite the promising outcomes, this study has several limitations. Given the exploratory nature of the study, only a single intranasal mAb dose (20 µg) and pretreatment interval (1 h) were evaluated. While effective, this precludes assessment of the minimal effective dose, optimal timing, or duration of protection. Ongoing studies are exploring dosing (5–50 µg) and administration times (30 min to 24 h) to optimize therapeutic durability. The study was conducted exclusively in a murine model, which may not fully replicate the complexity and heterogeneity of human allergic disease. The number of animals per group (n = 5) was limited in accordance with the approved ethical protocol and the principle of reduction - one of the core tenets of the 3Rs (Replacement, Reduction, and Refinement) guiding animal research. While sufficient for preliminary proof-of-concept, this sample size limited the statistical power and generalizability of the findings. Future studies should include larger cohorts to ensure reproducibility and enable more robust statistical comparisons. A key limitation is the lack of the mechanism of XA19 action. In particular, it remains unclear how a mAb recognising a single recombinant Art v 1 epitope can block a much broader allergenic response induced by native pollen which contains multiple allergenic proteins. Although the observed clinical and immunological improvements suggest pollen allergen neutralization, apart from showing a modest competive inhibition of IgE-allergen binding we do not know how XA19 interferes with IgE-FcϵRI binding or otherwise prevents mast cell/basophil degranulation. However, our structural modelling studies did suggest potential additive or alternative mechanisms whereby binding of XA19 to the defensin-like head domain of Art v 1 which has been shown to be target of human IgE antibodies, prevents their binding by direct steric hindrance, or by its binding inducing a conformational change in Art v 1 that in turn results in a shielding or a loss of the relevant IgE eptitopes.

Future studies involving mediator release assays (e.g., β-hexosaminidase, CD63/CD203c upregulation), and receptor occupancy analyses are warranted to clarify the mechanism. Notably, similar allergen-specific mAbs (e.g., Fel d 1–specific IgG) have been shown to prevent FcϵRI crosslinking without altering systemic IgE levels (15). The lower inhibition by XA19 of IgE binding to pollen extract versus recombinant Art v 1 may be attributed to a greater number of IgE epitopes within the complex mixture due to IgE recognition of other allergens. Nevertheless, XA19 was still able to suppress clinical symptoms induced by pollen extract challenge. Another limitation is that the study did not include an irrelevant IgG_1_ isotype control to exclude nonspecific effects of intranasal antibody administration. Future studies will need to incorporate such controls to confirm specificity. Histopathological evaluation relied on semi-quantitative scoring and although standardized, these methods are subjective. Future studies will need to incorporate digital morphometric analysis (e.g., eosinophils/mm², goblet cell density) to improve quantification. The study did not assess upstream epithelial-derived cytokines IL-33 or TSLP, which are pivotal in type 2 inflammation and we are validating assays to include these markers in future experiments. Airway hyperresponsiveness was assessed using Penh, a widely used but indirect marker. Its limitations are recognized. Future validation studies will employ invasive plethysmography (e.g., forced oscillation techniques) for precise quantification. Although no signs of local irritation or systemic distress were noted, formal toxicity evaluations including histopathological scoring and cytokine profiling (e.g., IL-6, TNF) were not performed and are planned for future studies. A key limitation of the present study is the absence of a direct comparison between intranasally administered mAb and either parenteral passive AIT or conventional AIT using subcutaneous or sublingual routes. Although injectable allergen-blocking mAbs have shown clinical efficacy, intranasal delivery may offer advantages in mucosal targeting, safety, and patient compliance. Future studies should include direct comparisons to determine whether local administration provides comparable or superior outcomes. Notably, our recent study comparing SCIT and SLIT with recombinant Art v 1 in the same murine model demonstrated marked reductions in airway inflammation and Th2 cytokines (21), which can serve as a useful benchmark for further development of intranasal passive AIT approaches. Although the results suggest that mAb XA19 exerts its effect independently of systemic IgE levels, we did not perform basophil activation tests (BAT) or receptor occupancy assays to confirm direct inhibition of FcϵRI-mediated effector cell degranulation. We are currently optimizing BAT protocols using human basophils from sensitized donors, stimulated with mugwort allergens in the presence or absence of XA19, to assess CD63/CD203c upregulation. These studies should provide important mechanistic insights. The long-term immunological effects of repeated intranasal mAb administration were not assessed as the study focused on acute allergic responses. It remains unclear whether allergen-specific mAbs can modulate long-term immune tolerance or disease progression. The present study did not test a humanized mAb with creation of a humanised version of XA19 being the next step needed to bridge the gap between preclinical findings and potential human therapeutic use. A limitation of intranasal delivery may be the rapid clearance of mAbs from mucosal surfaces, which may shorten the window of protection. This was exemplified in our prior experiments, where anti-SARS-CoV-2 mAbs administered intranasally to hamsters 8 h before viral challenge was not protective versus mAb given 1 h before challenge (unpublished data). To overcome this barrier, future studies should explore the development of mucoadhesive delivery systems to prolong mAb residence time on respiratory mucosa and enhance the durability of protection.

To conclude, our results demonstrate, for the first time, that intranasal delivery of XA19, an allergen-specific mAb confers robust protection against both upper and lower airway inflammation in a validated mouse model of *Artemisia*-induced allergy. The XA19 mAb pretreatment markedly reduced clinical symptoms, histological damage, ear swelling responses, AHR, and key Th2 cytokines (IL-4, IL-5), despite no impact on systemic IgE levels. These data underscore the promise of this allergen-specific therapeutic approach to aero-allergy and support its further development.

## Data Availability

The raw data supporting the conclusions of this article will be made available by the authors, without undue reservation.
